# A Guide to PIN1 Function and Mutations Across Cancers

**DOI:** 10.3389/fphar.2018.01477

**Published:** 2019-01-22

**Authors:** Maguie El Boustani, Lucia De Stefano, Isabella Caligiuri, Nayla Mouawad, Carlotta Granchi, Vincenzo Canzonieri, Tiziano Tuccinardi, Antonio Giordano, Flavio Rizzolio

**Affiliations:** ^1^Pathology Unit, IRCCS CRO Aviano-National Cancer Institute, Aviano, Italy; ^2^Doctoral School in Molecular Biomedicine, University of Trieste, Trieste, Italy; ^3^Doctoral School in Chemistry, University of Trieste, Trieste, Italy; ^4^Department of Pharmacy, University of Pisa, Pisa, Italy; ^5^Sbarro Institute for Cancer Research and Molecular Medicine, Center for Biotechnology, College of Science and Technology, Temple University, Philadelphia, PA, United States; ^6^Department of Molecular Science and Nanosystems, Ca’ Foscari University of Venice, Venice, Italy

**Keywords:** PIN1, cancer, mutations, SNP, 3D modeling

## Abstract

PIN1 is a member of a family of peptidylprolyl isomerases that bind phosphoproteins and catalyze the rapid *cis*–*trans* isomerization of proline peptidyl bonds, resulting in an alteration of protein structure, function, and stability. PIN1 is overexpressed in human cancers, suggesting it promotes tumorigenesis, but depending on the cellular context, it also acts as a tumor suppressor. Here, we review the role of PIN1 in cancer and the regulation of PIN1 expression, and catalog the single nucleotide polymorphisms, and mutations in *PIN1* gene associated with cancer. In addition, we provide a 3D model of the protein to localize the mutated residues.

## Introduction

Proline is the only amino acid with the ability to adopt either a *cis* or *trans* conformation, and this isomerization is catalyzed by peptidylprolyl isomerases (PPIases). The *cis*-*trans* isomerization of proline in phosphorylated Ser/Thr-Pro motifs is catalyzed exclusively by PIN1 (peptidylprolyl *cis/trans* isomerase, NIMA-interacting 1) (Liou et al., 2011).

PIN1-mediated isomerization is an important regulatory mechanism in human physiology and pathology: the conformational change regulates various protein functions, including the catalytic activity, the phosphorylation status, protein interaction, subcellular location, and/or protein stability (Lu et al., 1996).

Structurally, PIN1 has two domains connected by a flexible linker: the N-terminal domain is called “WW” (referring to two invariant Trp residues) and targets the enzyme to pSer/Thr-Pro motifs in substrates; the C-terminal PPIase domain has the catalytic activity (Lu et al., 1996).

PIN1 is involved in cellular processes such as the cell cycle, the folding of newly synthesized proteins, responses to DNA damage and stress, and immune responses (Lu et al., 1996). It is overexpressed in several human cancers (Lee et al., 2011), including prostate cancer (Ayala et al., 2003; La Montagna et al., 2012), breast cancer (Wulf et al., 2001; Ryo et al., 2002; Lucchetti et al., 2013), and oral squamous carcinomas (Miyashita et al., 2003). However, it is still not fully understood how this enzyme participates in cancer development and progression. Several studies showed that some single nucleotide polymorphisms (SNPs) in *PIN1* gene increase the risk of cancer whereas other variants operate as protective factors (Segat et al., 2007; Lu et al., 2009; Han et al., 2010; Li et al., 2013; Huang et al., 2016). Little has been reported so far about *PIN1* somatic mutations and cancer. This review summarizes the role of PIN1 in cancer and the regulation of PIN1 expression, and is an exhaustive guide to *PIN1* SNPs and mutations across cancers.

## Pin1 as an Oncogene or Conditional Tumor Suppressor Gene

*PIN1* has been shown to be a proto-oncogene whose protein product regulates several proteins involved in cancer initiation and progression (Zhou and Lu, 2016; Russo Spena et al., 2018). For example, PIN1 upregulates the expression of cyclin D1 at both the transcriptional and post-translational levels. At the transcriptional level, PIN1 activates transcription of the gene encoding cyclin D1 (*CCND1*) via two signal transduction pathways. In the Ras signaling pathway, activation of a kinase cascade leads to phosphorylation, and activation of JNK (c-Jun N-terminal kinase), which phosphorylates and activates the transcription factor c-Jun. PIN1 can bind and isomerize both phosphorylated JNK and phosphorylated c-Jun to potentiate c-Jun transcriptional activity at the *CCND1* promoter (Wulf et al., 2001).

PIN1 also stimulates cyclin D1 expression via the Wnt / β-catenin pathway. Briefly, in unstimulated cells, a complex composed of adenomatous polyposis coli (APC), glycogen synthase kinase 3β (GSK-3β), and other proteins keeps cytosolic levels of β-catenin low by triggering this protein’s phosphorylation, ubiquitination and degradation. When extracellular Wnt proteins activate their receptor (composed of a Frizzled receptor and other proteins), GSK-3β is displaced from the complex so β-catenin can accumulate and translocate to the nucleus. There, β-catenin binds transcription factors and other co-activators in a transcription complex that activates *CCND1* and other Wnt target genes (MacDonald et al., 2009). PIN1 and β-catenin levels are strictly correlated. PIN1 inhibits the APC-dependent exporting of β-catenin from the nucleus to the cytoplasm and cytoplasmic degradation of β-catenin, thereby β-catenin accumulates in the nucleus where it activates the transcription of genes such as *CCND1* (Ryo et al., 2001).

At the protein level, PIN1 isomerizes cyclin D1; this protein modification has a stabilizing effect (Liou et al., 2002). Cyclin D1 then accumulates in the nucleus, where in concert with other proteins it drives cell cycle progression (Liou et al., 2002; Ryo et al., 2002; Gladden and Diehl, 2005). The cyclin D1 activation as downstream target suggests that PIN1 coordinates different events of cell cycle, by acting as molecular timer, and that the overexpression of PIN1 in cancer leads to uncontrolled cell cycle.

Other oncogenic proteins stabilized by being isomerized by PIN1 are Akt (also called protein kinase B), retinoblastoma-associated protein (pRb), and myeloid cell leukemia 1 protein (MCL-1). PIN1 isomerization of Akt is critical for activation of the Akt signaling cascade that in turn activates the transcription of genes encoding cyclin D1, p53 and IKK-NFκB. In cancer cells, high levels of PIN1 amplify the activation of the Akt cascade and thus enhance tumor progression (Liao et al., 2009). PIN1 isomerization of pRb facilitates its binding to CDK–cyclin complexes in mid- to late G1. As a result, pRb is hyperphosphorylated and orchestrates cell proliferation by allowing the expression of genes that mediate entry into the S phase via the E2F transcription factor. In cancer, PIN1 overexpression leads to pRb pathway iperactivation (Rizzolio et al., 2012, 2013). Finally, isomerization of MCL-1 causes a conformational change that may stabilize the protein and enhance its anti-apoptotic function. Briefly, MCL-1 is phosphorylated by GSK-3β, facilitating MCL-1 association with the E3 ligase β-TrCP. The interaction between MCL-1 and the GSK-3β–E3 ligase β-TrCP complex leads to MCL-1 ubiquitination and degradation (Ding et al., 2007). PIN1-mediated isomerization may prevent MCL-1 association with the GSK-3β–E3 ligase β-TrCP complex, blocking MCL-1 degradation, but further studies are required (Ding et al., 2008).

Finally, PIN1 isomerizes two transcription factors, namely NF-κB, increasing its nuclear retention (Ryo et al., 2003), and STAT3, promoting its transactivation (Ryo et al., 2003; Lufei et al., 2007). These two proteins are involved in inflammation-induced carcinogenesis and are constitutively activated in several cancers (Grivennikov and Karin, 2010). PIN1’s action on these transcription factors enhances the transcription of genes encoding cyclin D1, c-Myc and Bcl-2 (Ryo et al., 2003; Lufei et al., 2007).

Altogether, these results feature PIN1 as a tumor promoter, but Yeh and Means described PIN1 as a “conditional” tumor suppressor (Yeh and Means, 2007) and successive studies support this theory. Indeed, PIN1 can induce apoptosis, prevent genomic instability, and promote the ubiquitin-dependent proteolysis of many oncogenic proteins. All these processes limit tumor progression.

In stress conditions, PIN1 induces apoptosis via p53 and p73 (Mantovani et al., 2004). Moreover, a study on murine embryonic fibroblasts showed that PIN1 prevents p53-dependent genomic instability (Wulf et al., 2004).

PIN1 is involved in the ubiquitin-dependent proteolysis of Myc, Bcl-6 and cyclin E (Yeh et al., 2004; Yi et al., 2005; Phan et al., 2007; Farrell et al., 2013). For example, PIN1 binds to doubly phosphorylated Myc on Thr58 and Ser62. The conformational change facilitates Myc dephosphorylation on Ser62 by protein phosphatase 2 (PP2A), which allows Myc ubiquitination and degradation by the proteasome (Farrell et al., 2013). Additionally, upon DNA damage, the kinase ATM phosphorylates Bcl-6 that in turn becomes a substrate for PIN1. Bcl-6 isomerization signals its degradation by the ubiquitin-proteasome system (Phan et al., 2007).

Finally, PIN1 regulates the degradation of cyclin E during the G0/G1-S phase transition of the cell cycle permitting to cell cycle to proceed correctly (Yeh et al., 2006). Experiments in which PIN1 expression was down-regulated showed increased steady-state levels of cyclin E, the arrest of cells in G1/S phase, genomic instability, and tumoral transformation (Yeh et al., 2006).

## Regulation Of PIN1 Expression

### Transcriptional Regulation

*PIN1* transcription can be activated by the E2F transcription factor or by Notch1 binding to the *PIN1* promoter (Ryo et al., 2002; Rustighi et al., 2009). In this way PIN1 sustains the transformed phenotype induced by E2F or Notch1 activation.

*PIN1* transcription can also be suppressed by the tumor suppressor gene *BRCA1* (MacLachlan et al., 2000). *BRCA1* associates with several proteins to regulate DNA repair response. In cancer, *BRCA1* is often mutated and lost such function, thereby cells accumulates DNA damage (Mersch et al., 2015).

Recently, micro-RNAs (miRNAs) have been identified as regulators of *PIN1* expression. For instance, miR200c binds to a conserved region in the 3′-untranslated region (UTR) of *PIN1* mRNA and prevents its translation (Luo et al., 2014). Mutations in this region of *PIN1* can prevent the repressive effects of miR200c (Luo et al., 2014). miRNA-200b, and miR-296-5p also bind the 3′ UTR of *PIN1* mRNA and down-regulate its expression. In cancer cells, both these miRNAs were found to be underexpressed, allowing PIN1 to sustain tumor progression (Zhang et al., 2013; Lee et al., 2014).

### Post-translational Regulation

Depending on the physiological or pathological conditions, the activity of proteins is regulated by post-translational modifications. For PIN1, there is evidence of post-translational modification by phosphorylation, ubiquitination, SUMOylation and oxidization at specific sites.

Phosphorylation of PIN1 on Ser16 in the WW domain suppresses its ability to interact with its substrates (Lu et al., 2002). At least three kinases can phosphorylate this residue: protein kinase A (Lu et al., 2002), ribosomal S6 kinase 2 (Cho et al., 2012), and aurora kinase A (Lee et al., 2013). Phosphorylation on Ser71 in the PPIase domain inhibits the protein’s enzymatic activity (Lee et al., 2013). Phosphorylation on Ser65 by polo-like kinase (Plk1) (Eckerdt et al., 2005) and on Ser138 by mixed-lineage kinase 3 increases PIN1’s catalytic activity and nuclear translocation (Rangasamy et al., 2012). Plk1-mediated Ser65 phosphorylation is suggested to regulate PIN1 turnover. It induced PIN1 deubiquitination and stabilization, while the absence of Plk1 enhanced the ubiquitination and degradation of PIN1 (Eckerdt et al., 2005).

SUMOylation of Lys6 in the WW domain and Lys63 in the PPIase domain abolishes PIN1’s enzymatic activity and oncogenic functions (Chen et al., 2013). However, deSUMOylation of these two domains by SUMO1/sentrin specific peptidase 1 (SENP1) restores PIN1’s activity. SENP1 overexpression increases the levels of deSUMOylated PIN1 and in turn the ability of PIN1 to induce centrosome amplification and cell transformation (Chen et al., 2013).

Finally, under conditions of oxidative stress, PIN1 is oxidized on Cys113 in the catalytic site, inhibiting its enzymatic activity (Chen et al., 2015).

### PIN1 Single Nucleotide Polymorphisms and Cancer Risk

Several SNPs, located in the promoter or coding region of *PIN1*, are associated with cancer risk. The *PIN1* variants rs2233678 (c.–842G>C) and rs2233679 (c.–667T>C), both located in the promoter, and the synonymous change rs2233682 (G>A; p.Gln33Gln) in exon 2 of the coding region, have been widely investigated.

The −842C allele of rs2233678 was found to confer a significantly lower risk of cancer (odds ratio, 0.75) in a meta-analysis of 11 studies (9280 participants) of patients with esophageal carcinoma, nasopharyngeal carcinoma, laryngeal squamous cell carcinoma, lung cancer, breast cancer, squamous cell carcinoma of the head, and neck or hepatocellular carcinoma (HCC), and matched healthy controls (Li et al., 2013). Seven of the included studies had found that the allele reduced risk, while four found no association with cancer risk.

A study of 209 patients with oral squamous cell carcinoma and 444 controls did not find an association between the −842G>C polymorphism and cancer risk (Yao et al., 2014).

The −667T>C polymorphism has been found to not associate with esophageal carcinoma (You et al., 2013), breast cancer (Han et al., 2010), or squamous cell carcinoma of the head and neck (Lu et al., 2009), whereas it did associate with a lower risk of nasopharyngeal carcinoma (Lu et al., 2013), and a higher risk of oral squamous cell carcinoma (Yao et al., 2014).

Finally, the synonymous change Gln33Gln was not found to associate with the risk of breast cancer (Han et al., 2010), or squamous cell carcinoma of the head and neck (Lu et al., 2009). However, a higher risk of HCC was found among carriers of the Gln33Gln variant in a Chinese population (Huang et al., 2016).

### PIN1 Somatic Mutations in Cancer Tissues

Because of the lack of published papers reporting on *PIN1* somatic mutations, we obtained deposited genetic data on these mutations in different tumor types from the cBioPortal for Cancer Genomics and COSMIC (Catalog of Somatic Mutations in Cancer). cBioPortal is the main resource for the analysis of large-scale cancer genomics datasets (Cerami et al., 2012; Gao et al., 2013). COSMIC, a database of mutations reported in the scientific literature or from the Cancer Genome Project, permits researchers to explore the effects of somatic mutations in cancer (Forbes et al., 2017).

The data obtained from cBioPortal regarded 11,000 cancer cases collected for genomic characterization in December 2013 and 32,555 cases in 61 primary sites of cancer (retrieved on June 13, 2018) (Cerami et al., 2012; Gao et al., 2013). The data from COSMIC refer to 41,924 unique samples from patients with different types of cancer including skin, breast, intestinal, lung, liver, prostate, and stomach cancer (retrieved on June 14, 2018) (Forbes et al., 2017). Altogether, the data revealed the existence of 32 somatic mutations affecting 29 unique residues in the coding region of *PIN1* gene (Table 1). Five mutations affect the WW domain (residues 1–39), two are in the flexible linker (residues 35–53), and 25 affect the PPIase domain (residues 50–163). Twenty-three are missense mutations, four are synonymous mutations (they have no effect on PIN1 function), and four are nonsense mutations (R21^∗^, S71^∗^, S108^∗^, and E163^∗^). Overall, 17 mutations were predicted to be pathogenic by the Functional Analysis through Hidden Markov Models (FATHMM) filter in COSMIC and three to be deleterious by the Sorting Intolerant from Tolerant (SIFT) algorithm in cBioPortal. The others are predicted to be tolerated.

**Table 1:** *PIN1* mutations in cancer.

AA change	Type	Predicted functional consequence	Position (GRCh37)	Nucleotide change	Cancer	Patients n^§^	Frequency %	Reference or study identifier
G20G	Splicing	None^a^	9949113	C>T	Skin cutaneous melanoma^1^	121	0.83	Hodis et al., 2012
R21^∗^	Nonsense	Pathogenic^b^	9949114	C>T	Skin cutaneous melanoma^2^	366	0.27	Krauthammer et al., 2015
Q33K	Missense	Pathogenic^b^	9949150	C>A	Skin^2^	1215	0.08	Durinck et al., 2011
R36P	Missense	Pathogenic^b^	9949160	G>C	Large intestine^2^	1482	0.07	The Cancer Genome Atlas [TCGA], 2012
G39C	Missense	Deleterious^a^	9949168	G>T	HCC^3^	373	0.54	Cerami et al., 2012; Gao et al., 2013
G39C	Missense	Pathogenic^b^	9949168	G>T	SCLC^2^	42	2.38	Rudin et al., 2012
S42I	Missense	None^b^	9949178	G>T	Large intestine^2^	1482	0.07	Tahara et al., 2014
Q49Q	Synonymous	None^b^	9949200	G>A	Skin^2^	1215	0.08	COSU540^c^
V55I	Missense	Pathogenic^b^	9949216	G>A	ER^+^ breast cancer^2^	2103	0.05	Robinson et al., 2013
S71*	Nonsense	None^a^	9949265	C>A	Sarcoma^3^	247	0.40	Cerami et al., 2012; Gao et al., 2013
S71L	Missense	Pathogenic^b^	9949265	C>T	Skin^2^	1215	0.08	Pickering et al., 2014
S71S	Synonymous	Neutral^b^	9949266	G>A	Stomach adenocarcinoma^2^	790	0.13	COSU541^c^
E100D	Missense	Tolerated^a^	9958734	G>T	CRC^4^	224	0.45	The Cancer Genome Atlas [TCGA], 2012
E104K	Missense	Tolerated^a^	9958744	G>A	NSCLC^5^	1144	0.09	Campbell et al., 2016
S105F	Missense	None^b^	9958748	C>T	Large intestine^2^	1482	0.07	The Cancer Genome Atlas [TCGA], 2012
S108^∗^	Nonsense	Pathogenic^b^	9958757	C>A	Skin^2^	1215	0.08	Durinck et al., 2011
D112N	Missense	Pathogenic^b^	9958768	G>A	Large intestine^2^	1482	0.07	The Cancer Genome Atlas [TCGA], 2012
A124V	Missense	Pathogenic^b^	9958805	C>T	Stomach adenocarcinoma^2^	289	0.35	COSU541^c^
P133L	Missense	Pathogenic^b^	9959781	C>T	Desmoplastic melanoma^2^	20	5.00	Shain et al., 2015
F134S	Missense	Deleterious^a^	9959784	T>C	Neuroendocrine prostate cancer^6^	81	1.23	Beltran et al., 2016
S138S	Synonymous	Neutral^b^	9959797	G>A	Large intestine^2^	1482	0.07	Giannakis et al., 2014
F139S	Missense	Pathogenic^b^	9959799	T>C	Cervical squamous cell carcinoma^2^	194	0.52	COSU415^c^
T143M	Missense	Deleterious^a^	9959811	C>T	Adeno-cortical carcinoma^3^	90	1.11	Cerami et al., 2012; Gao et al., 2013
G144E	Missense	Pathogenic^b^	9959814	G>A	HCC^2^	1816	0.06	COSU381^c^
E145K	Missense	Tolerated^a^	9959816	G>A	Head & neck squamous cell carcinoma^3^	510	0.20	Cerami et al., 2012; Gao et al., 2013
G148R	Missense	Pathogenic^b^	9959825	G>C	Esophagus-stomach cancers^2^	518	0.19	Cancer Genome Atlas Research Network et al., 2014
G148G	Synonymous	None^b^	9959827	G>A	Biliary tract cancer^2^	366	0.27	COSU658^c^
P149S	Missense	Pathogenic^b^	9959828	C>T	Skin^2^	1215	0.08	Wei et al., 2011
T152M	Missense	Pathogenic^b^	9959838	C>T	CRC^2^	619	0.16	Giannakis et al., 2016
S154F	Missense	Deleterious^a^	9959844	C>T	NSCLC^5^	1144	0.09	Campbell et al., 2016
H157Y	Missense	Pathogenic^b^	9959852	C>T	Skin^2^	1215	0.08	Nikolaev et al., 2011
T162I	Missense	Pathogenic^b^	9959868	C>T	Large intestine^2^	1482	0.07	Mouradov et al., 2014
E163*	Nonsense	None^b^	9959870	G>T	Squamous cell carcinoma^2^	1835	0.05	COSU583^c^


*^§^Number of patients in cancer study; ^∗^The mutation inserts a stop codon; ^*a*^SIFT algorithm in cBioPortal; ^*b*^FATHMM filter in COSMIC; ^*c*^COSMIC study identifier; ^*1*^Skin cutaneous melanoma-cBioPortal; ^*2*^COSMIC database; ^*3*^TCGA, PanCancer Atlas-cBioPortal; ^*4*^TCGA, Colorectal Adenocarcinoma-cBioPortal; ^*5*^Pan-lung cancer-cBioPortal; ^*6*^Neuroendocrine Prostate cancer-cBioPortal (Trento/Cornell/Broad). CRC, colorectal cancer; HCC, hepatocellular carcinoma; NSCLC, non-small cell lung cancer; SCLC, small cell lung cancer.*

**FIGURE 1:**
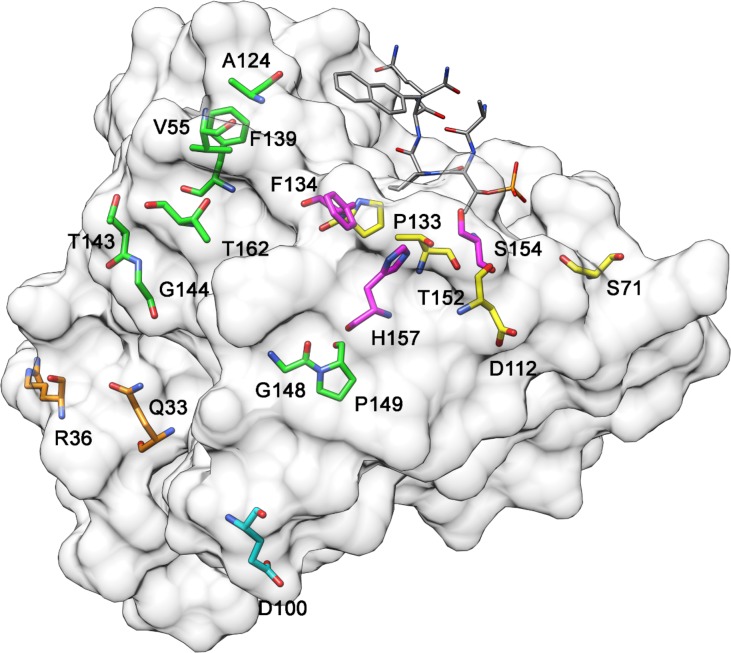
3D model of PIN1 protein highlighting amino acids that have undergone pathogenic mutation in cancer cases. *Orange*, mutated residues in the WW domain; *magenta*, residues in the PPIase domain that interact directly with substrates; *yellow*, residues in the PPIase domain that interact indirectly with substrates; *green*, residues in the PPIase domain that do not interact with substrates The gray molecule is a non-natural peptide inhibitor bound to PIN1 in the X-ray crystal structure (Protein Data Bank accession code, 2ITK) (Zhang et al., 2007).

Figure 1 illustrates the positions of PIN1 somatic mutations that alter the protein’s primary sequence and are predicted to be pathogenic or deleterious. This model is based on the X-ray crystal structure of PIN1 bound to a non-natural peptide inhibitor (Protein Data Bank accession code, 2ITK) (Zhang et al., 2007). The Q33K and R36P mutations (orange) are in the WW domain, as is G39C that is not shown because it belongs to a peptide loop missing from the X-ray structure. All the other mutations are found in the PPIase domain: F134S, S154F, and H157Y (magenta) interact with the enzyme’s substrates (Ranganathan et al., 1997; Wilson et al., 2013) while S71L, D112N, P133L, and T152M (yellow) are indirectly involved in the interaction with substrates (Ranganathan et al., 1997; Behrsin et al., 2007; Namanja et al., 2011). The other mutations (green) do not interact with substrates and thus could have a role in interactions with other proteins or in PIN1 protein folding. Among them, F139S is within the PPIase domain interface (S138 to R142) that is involved in interdomain communication and regulates the function of PIN1 upon substrate binding (Behrsin et al., 2007; Namanja et al., 2011).

### PIN1 Inhibitors

**FIGURE 2:**
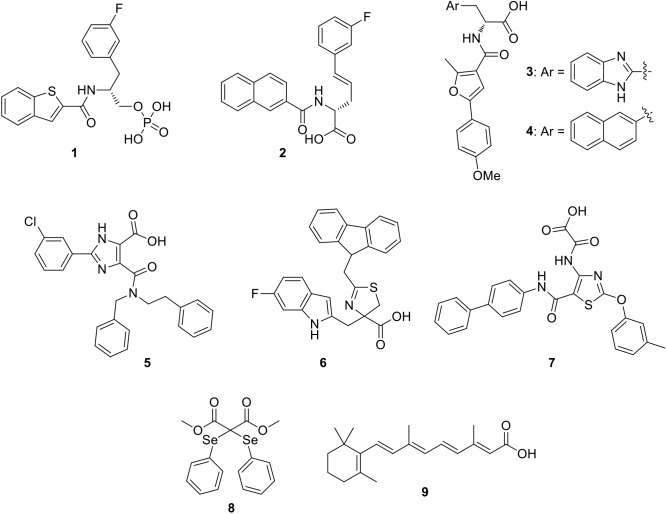
Most representative PIN1 inhibitors.

In the last decade, some PIN1 inhibitors were discovered by industries and academic research groups. Compounds **1** (Guo et al., 2009) and **2** (Dong et al., 2010; Figure 2), developed by Pfizer Worldwide Research & Development, are nanomolar PIN1 inhibitors (*K*_i_ = 6 and 890 nM for compounds **1** and **2**, respectively), as confirmed by X-ray structures that showed their binding to the enzyme. Unfortunately, both of them failed to be active in cell-based assays, likely due to the phosphate or carboxylate groups which were necessary to properly interact in a charged pocket of the enzyme, but strongly limited cell membrane permeability.

The α-Amino acid-derived compounds **3** and **4** (Figure 2) were discovered by researchers at Vernalis (R&D) Ltd. (Potter A.J. et al., 2010). These compounds maintained the carboxylate group, which is the moiety necessary for an optimal interaction in the enzyme binding site, and the aromatic portions were varied, inserting benzimidazole (**3**), or naphthalene (**4**), since they lied in a hydrophobic region of the protein. Compound **3** showed an IC_50_ value in the submicromolar range (0.13 μM), but it was unable to be active in cellular assays. Nevertheless, with the aim of reducing the polar surface area, its analog **4** was about twenty-fold less potent on the isolated enzyme (IC_50_ = 2.6 μM), but it gained activity in PC3 prostate cancer cells in which it reduced the proliferation.

Due to the problems encountered with the benzimidazole/naphthyl-based compounds, a further development in the search for PIN1 inhibitors at Vernalis (R&D) Ltd, consisted in the phenyl-imidazole derivatives. As a result, compound **5** (Figure 2) reached an optimal balance between inhibition activity on PIN1 (IC_50_ = 0.83 μM) and antiproliferative activity in PC3 cells (GI_50_ = 13 μM) (Potter A. et al., 2010).

The dihydrothiazole series, exemplified by compound **6** (Figure 2), was discovered in 2014 at Pfizer Worldwide Research & Development: in this chemical class, the amide group present in all the PIN1 inhibitors previously reported in literature was substituted by a dihydrothiazole ring, bearing a carboxylic acid moiety, with aim of decreasing polar surface area, which is a factor strongly influencing cellular permeability (Asso et al., 2008; Mikami et al., 2017). They showed micromolar inhibition potencies (IC_50_ values ranging from 1.9 to 27 μM) on PIN1 and they were able to reduce the proliferation of colon cancer cells (HT29) (Guo et al., 2014).

A new class of PIN1 inhibitors developed by Zhao et al. consisted in thiazole derivatives bearing oxalic or acetic acid group at 4-position and, according to modeling studies, this last portion was found to be located in the charged pocket of the enzyme. Compound **7** (Figure 2) showed an IC_50_ value of 2.93 μM, but unfortunately no data about its activity in cancer cells was reported (Zhao et al., 2016). Among the recently developed PIN1 inhibitors, a selenium containing compound (compound **8**, Figure 2) was identified by a novel high-throughput screening study. Compound **8** efficiently inhibited PIN1 (IC_50_ = 0.43 μM) and it was able to affect the proliferation of breast MDA-MB-231 cancer cells in which PIN1 is overexpressed, also reducing the viability of induced cancer stem cell-like cells (Subedi et al., 2016).

Wei et al. (2015) identified PIN1 as a target of all-trans retinoic acid (ATRA), compound **9** (Figure 2). ATRA inhibited and degraded PIN1 (*K*_i_ value of 0.82 μM), as confirmed by the co-crystal structure of ATRA with PIN1. Furthermore, ATRA was able to suppress the growth of triple-negative breast cancer and acute promyelocytic leukemia cells, both in humans and in animal models.

**FIGURE 3:**
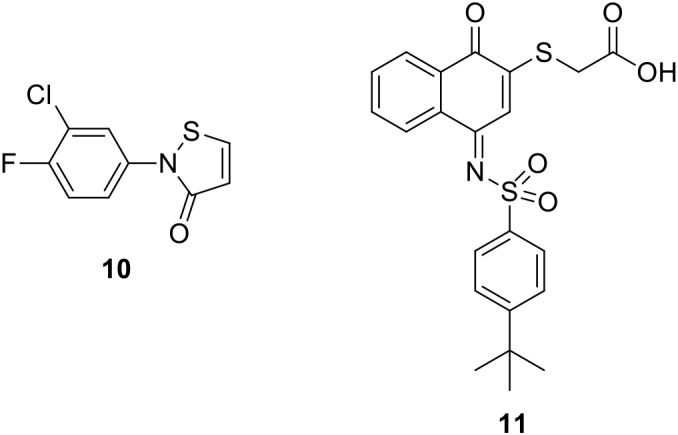
Covalent PIN1 inhibitors.

It is also noteworthy to mention two covalent PIN1 inhibitors. The α,β-unsaturated isothiazolone derivative **10** (Figure 3) showed a micromolar inhibition activity on the enzyme (IC_50_ = 6.1 μM); however, it was not selective for PIN1 also exerting a similar activity on cyclophilin (IC_50_ = 13.7 μM) (Mori et al., 2011). The quinone-sulfonamide derivative **11** (Figure 3) covalently bound Cys 113 (IC_50_ value of 0.64 μM), ultimately leading to PIN1 degradation. Moreover, **11** impaired PIN1-dependent invasive behavior of breast (MDA-MB-231) and prostate (PC3) cancer cells (Campaner et al., 2017).

## Conclusion

PIN1 is overexpressed in various cancer types and is associated with a malignant phenotype and tumor progression (Bao et al., 2004; Yeh and Means, 2007; Zhou and Lu, 2016). PIN1 may also have an anti-cancer role depending on the cellular context; therefore PIN1 has been called a conditional tumor suppressor gene (Yeh et al., 2006; Yeh and Means, 2007). Some SNPs in *PIN1* gene were found to associate with cancer risk. Carriers of the −842C variant in the *PIN1* promoter have low PIN1 protein levels and low risk for developing cancer (Li et al., 2013). Contrasting evidence has been reported for the −667C variant in the *PIN1* promoter, which was found to associate with a low risk of developing nasopharyngeal carcinoma (Li et al., 2013), but a high risk for oral squamous cell carcinoma and HCC (Yao et al., 2014; Huang et al., 2016).

So far, 32 somatic mutations in *PIN1* gene have been found in different types of cancer. Of these, 20 are predicted to be pathogenic or deleterious. Although further studies are required, we believe that investigating the complex pattern of *PIN1* gene alterations and their effects on PIN1 protein structure and function is a valid strategy for identifying new biomarkers for susceptibility to cancer and response to anti-PIN1 inhibitors.

## Author Contributions

All the authors wrote and approved the manuscript.

## Conflict of Interest Statement

The authors declare that the research was conducted in the absence of any commercial or financial relationships that could be construed as a potential conflict of interest.
